# Clinical Workload, Demographic Patterns, and Correlations in Neurology Ambulatory Care: A Single-Center Study from Bulgaria

**DOI:** 10.3390/ijerph23050651

**Published:** 2026-05-14

**Authors:** Christiyan Kirilov Naydenov, Antoaneta Petrova Yordanova

**Affiliations:** 1 Neurology Department, MHAT-MK “St. Iv. Rilsky”, Gerasim Papazchev 1 Street, 6000 Stara Zagora, Bulgaria; 2 Department of Social Medicine, Health Management and Disaster Medicine, Medical Faculty, Trakia University, Armeyska 11 Street, 6000 Stara Zagora, Bulgaria; antoaneta.yordanova@trakia-uni.bg

**Keywords:** ambulatory care, neurology, epidemiology, outpatient, Bulgaria

## Abstract

**Highlights:**

**Public health relevance—How does this work relate to a public health issue?**

- In this study, we address the growing clinical workload of neurological disorders as a major contributor to disability and healthcare utilization, with a specific focus on ambulatory care services in an aging population.
- We provide real-world evidence from an Eastern European setting, where detailed epidemiological data on ambulatory neurology services remain limited.

**Public health significance—Why is this work of significance to public health?**

- We identify substantial age-related patterns and diagnostic concentration within ambulatory neurology care, highlighting the dominance of a limited number of chronic neurological conditions.
- We demonstrate that diagnostic category, rather than gender or visit type, is the primary determinant of age distribution, emphasizing disease-specific clinical workload within outpatient care systems.

**Public health implications—What are the key implications or messages for practitioners, policy makers and/or researchers in public health?**

- We highlight the need for diagnosis-specific ambulatory care pathways and targeted resource allocation to address neurological conditions with high clinical workload, particularly in aging and resource-constrained healthcare systems.
- We provide region-specific epidemiological evidence to support health policy planning, improve outpatient neurology service efficiency, and facilitate benchmarking across healthcare systems.

**Abstract:**

Background: Neurological disorders are a leading cause of disability worldwide, placing increasing strain on healthcare systems. In Eastern Europe, and specifically Bulgaria, there is a significant lack of granular data regarding how ambulatory neurology services are utilized and how clinical workloads are distributed across different diagnostic groups. Objective: In this study, we aimed to analyze the clinical workload, demographic patterns, and diagnostic distribution within a single-center ambulatory neurology setting in Bulgaria, while identifying the primary determinants of patient age stratification. Methods: We conducted a retrospective observational study of 518 consecutive clinical encounters recorded over a one-year period in a specialized outpatient neurology clinic. Data on age, gender, visit type (ambulatory vs. dispensary), and ICD-10 diagnostic groups were analyzed. Inferential analyses included a one-way ANOVA for age differences and multivariable linear regression to identify independent predictors of age patterns, with age modeled as a continuous variable. Results: The clinical workload was highly concentrated, with spine-related disorders accounting for over 40% of all visits, and primary consultative examinations were the predominant service type (65.4%). Statistical analysis revealed significant age differences across diagnostic categories (*p* < 0.001), with neurodegenerative and cerebrovascular diseases associated with the highest mean age, while spine and headache syndromes involved significantly younger populations. Multivariable modeling confirmed that diagnostic category is the sole independent determinant of age distribution (*p* < 0.001), whereas gender and visit type showed no significant independent associations. Conclusions: Ambulatory neurology utilization in this setting is characterized by a high-turnover primary consultation model and a heavy concentration of musculoskeletal neurological conditions. These findings suggest that outpatient neurology functions as a critical diagnostic filter and pain management hub. The study underscores the need for diagnosis-specific clinical pathways and targeted resource allocation to optimize service efficiency and improve long-term management of chronic neurological morbidity in a public insurance-driven framework.

## 1. Introduction

According to recent global estimates, neurological conditions account for a substantial and increasing proportion of years lived with disability, particularly in aging populations [1,2]. Advances in acute neurological care and improved survival have further shifted the clinical workload toward chronic disease management, making ambulatory neurology services a central component of modern healthcare systems.

Ambulatory neurological care plays a critical role in the diagnosis, monitoring, and long-term management of a wide spectrum of conditions, including cerebrovascular disease, headache disorders, neurodegenerative diseases, and peripheral nervous system disorders. Previous studies from Western Europe and North America have demonstrated that neurology ambulatory clinics are characterized by high patient volumes, recurrent visits, and a diagnostic profile dominated by a limited number of chronic conditions [3,4,5]. These patterns have important implications for workforce planning, service organization, and health system sustainability but they remain limited in Eastern Europe, particularly in Bulgaria. This lack of granular ambulatory data constrains evidence-based planning and limits opportunities for benchmarking against international practice.

Age and sex are well-established determinants of neurological disease distribution and healthcare utilization. While population-level studies consistently demonstrate strong age dependence for many neurological conditions, less is known about how age-related patterns manifest within real-world ambulatory neurology practice. Furthermore, the extent to which demographic factors independently influence visit characteristics after accounting for diagnostic profiles remains insufficiently explored in ambulatory settings.

Understanding the interaction between demographic characteristics, diagnostic categories, and visit structure is essential for optimizing ambulatory neurology services. Such analyses can identify patient groups with a high clinical workload, inform the development of diagnosis-specific care pathways, and support more efficient allocation of healthcare resources.

In contrast to traditional epidemiological models that treat age primarily as a biological risk factor, we utilize age in the present study as the dependent variable in a multivariable analysis. This analytical approach is strategically chosen to characterize the demographic ‘fingerprint’ of the outpatient workload. By modeling age as a continuous outcome, we can precisely quantify how specific diagnostic categories and visit types independently dictate the age composition of the clinical flow. This shift in perspective is essential for health services research, as it treats age as a proxy for service demand and resource intensity, allowing for a clearer understanding of which clinical segments drive care utilization among different demographic strata.

To address the lack of studies utilizing granular visit-level data and multivariable analyses within real-world ambulatory environments, the aim of the present study was twofold: to describe the clinical workload of a Bulgarian ambulatory neurology center by mapping diagnostic distribution and patient demographics across distinct visit categories, and to test the independent associations between diagnostic category, gender, and visit type in determining the age profile of the clinical workload, using multivariable analytical approaches to clarify the unique contribution of each factor within this specific health system context.

## 2. Materials and Methods

### 2.1. Study Design and Setting

This study was conducted as a retrospective observational epidemiological analysis of ambulatory neurology visits in a single specialized neurology outpatient private center in Bulgaria, and it covered a one-year period (1 August 2024–30 July 2025) and was based on routinely collected clinical data. The Bulgarian outpatient healthcare system operates under a two-tier model regulated by the National Health Insurance Fund (NHIF), where clinical encounters are functionally classified into ambulatory and dispensary (follow-up for chronic conditions) visits. Ambulatory visits are designed for acute diagnostic episodes, where the primary visit initiates the care cycle and the secondary visit provides follow-up within a 30-day window. In parallel, dispensary observation is structured for the long-term monitoring of chronic diseases; the primary dispensary visit of the calendar year establishes the annual therapeutic plan, while subsequent secondary dispensary visits are conducted according to standardized intervals specific to the disease entity. This defined hierarchy of medical encounters provides a methodological framework for tracking patient flow and ensures continuity of care between primary and specialized outpatient sectors.

### 2.2. Inclusion and Exclusion Criteria

#### 2.2.1. Inclusion Criteria

To ensure a comprehensive mapping of the clinical workload, we applied a broad inclusion strategy. Eligible records met the following criteria:

Temporal: All clinical encounters recorded within the specified one-year study period.

Setting: Visits conducted exclusively within the designated ambulatory neurology center.

Data Integrity: Electronic health records (EHRs) contained complete information regarding the primary diagnostic category (ICD-10 code), patient age, gender, and visit type.

#### 2.2.2. Exclusion Criteria

To minimize selection bias and preserve the “real-world” nature of the utilization data, no formal exclusion criteria were applied at the outset. However, the following cases were considered ineligible for analysis:

Duplication: Duplicate entries for the same administrative encounter.

Incompleteness: Records with missing or unidentifiable diagnostic codes (note: in this cohort, 100% of records met the data integrity requirements, resulting in zero exclusions).

Outside Scope: Visits not covered by the National Health Insurance Fund (NHIF) framework if they did not follow the standardized ambulatory/dispensary visit structure.

### 2.3. Data Sources and Variables

Data were extracted from the electronic medical records of the ambulatory clinic and anonymized prior to analysis. The following variables were included:

### 2.4. Demographic Variables: Age (Years) and Gender

Visit characteristics: The type of ambulatory visit, categorized according to the clinic’s internal classification system.

Clinical variables: Predefined diagnostic groups were based on the internationally standardized coding system ICD-10.

Age was analyzed as a continuous variable, while gender, visit type, and diagnostic group were treated as categorical variables.

### 2.5. Statistical Analysis

Descriptive statistics were used to summarize the study population. Age was treated as a continuous variable for all inferential analyses to preserve statistical power and prevent information loss. For descriptive purposes and to visualize the sample distribution, age is additionally presented in a priori defined 10-year categories. Continuous variables are reported as means with standard deviations (SDs), medians, and ranges, while categorical variables are expressed as absolute frequencies and percentages.

To examine the primary research questions, a one-way analysis of variance (ANOVA) was employed to compare the mean age across different diagnostic groups. Upon detecting significant global differences, post hoc pairwise comparisons were conducted using Tukey’s Honestly Significant Difference (HSD) test. The magnitude of these associations was quantified using eta-squared (η^2^).

The independent contribution of clinical and demographic factors to age variance was assessed via multivariable linear regression, where age (continuous) served as the dependent variable, and diagnostic group, gender, and visit type were entered as independent predictors. Model fit was evaluated using the adjusted R^2^ coefficient. All tests were two-sided, with a significance threshold of *p* < 0.05.

### 2.6. Ethical Considerations

The study was conducted in accordance with the principles of the Declaration of Helsinki. Informed consent was not required, as the analysis was based on anonymized retrospective data collected during routine clinical care.

## 3. Results

### 3.1. Study Population and Visit Characteristics

A total of 518 ambulatory visits were included in the analysis over the one-year study period, with the study population consisting of 287 females (55.4%) and 231 males (44.6%). The age distribution showed a predominance of older adults, with the largest proportion observed in the group of patients aged 71–80 years (26.4%), followed by 61–70 years (15.1%) and 51–60 years (16.0%) (Table 1). The mean age was 56.9 ± 18.4 years, with a median of 59 (42.0–73.0) years, with a predominant concentration in the 7th and 8th decades of life (over 40% of patients were aged 61–80). This confirms that the outpatient workload is heavily skewed toward an aging population, which typically presents with higher diagnostic complexity and multi-morbidity.

**Figure 1:**
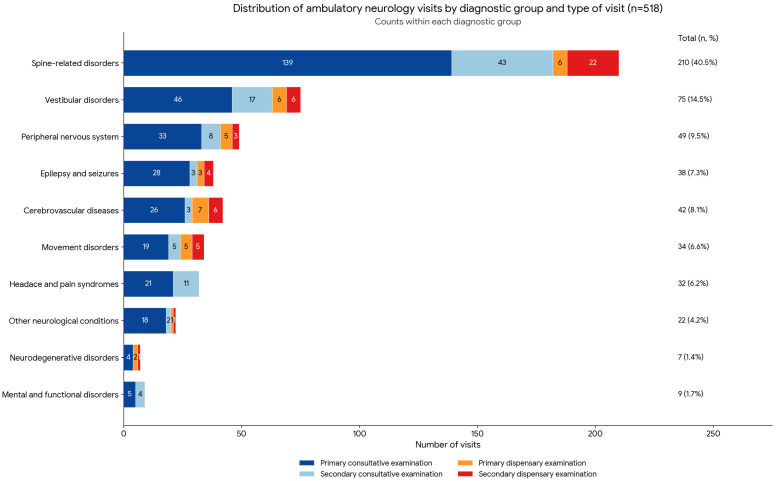
Composition of the clinical workload by diagnostic category and visit type.

Regarding the type of visit, primary consultative examinations accounted for the majority of visits (65.4%), followed by secondary consultative examinations (18.5%), secondary dispensary examinations (9.3%), and primary dispensary examinations (6.8%) (Table 1, Figure 1).

### 3.2. Diagnosis Group and Type of Visit

The distribution of visit types across diagnostic categories is presented in Figure 1.

The largest diagnostic category was spine-related disorders, representing 210 visits, followed by vestibular disorders (75 visits), peripheral nervous system disorders (49 visits), and epilepsy and seizures (38 visits). In all diagnostic groups, primary consultative examinations were the most frequent type of visit, ranging from 57.6% in movement disorders to 81.8% in other neurological conditions.

The association between diagnosis group and type of visit showed a borderline statistical significance (Pearson’s χ^2^ = 38.84, df = 27, *p* = 0.066), not reaching the predefined threshold of *p* < 0.05, with a small-to-moderate effect size (Cramer’s V = 0.158). These findings suggest heterogeneity in visit structure across diagnostic categories, while indicating that the overall pattern is dominated by primary consultative care.

### 3.3. Age Differences Across Diagnostic Groups

The significant age variation across diagnostic groups (F = 13.02, *p* < 0.001), with a large effect size (η^2^ = 0.19), demonstrates strong age-specific ‘clustering’ of neurological diseases. Specifically, while neurodegenerative and cerebrovascular cases were concentrated among the elderly, spine and headache syndromes predominantly affected the working-age population. This stratification is crucial for workforce planning, as it highlights the different socio-economic impacts of these diagnostic groups. Age differed significantly across diagnostic categories, with diagnostic group explaining approximately 19% of the variance in patient age (Table 2).

Post hoc analysis using Tukey’s HSD test revealed multiple statistically significant pairwise age differences between diagnostic groups, confirming substantial heterogeneity in age distribution (Table 3).

### 3.4. Association Between Gender and Visit Type

No significant association was observed between patient gender and type of ambulatory visit (χ^2^ = 1.57, *p* = 0.665), and the corresponding effect size was negligible (Cramér’s V = 0.055).

### 3.5. Multivariable Linear Regression Analysis

A multivariable linear regression model was constructed with age (years) as the dependent variable and diagnostic group, gender, and type of ambulatory visit as independent variables (Table 4).

The multivariable model explained approximately 20% of the variance in patient age (Adjusted R^2^ = 0.177). The finding that diagnostic category remained the only significant independent predictor (after adjusting for gender and visit type) confirms that clinical diagnosis is the primary driver of demographic patterns in this setting. Gender-neutral utilization patterns suggest that the healthcare system provides equitable access to both sexes for specific neurological conditions.

After adjustment for gender and visit type, diagnostic group remained independently associated with age. Spine-related, headache, and pain syndromes were associated with a significantly lower mean age compared with the reference group, with adjusted differences ranging from −7.8 to −26.5 years. The largest age differences were observed for neurodegenerative, motor, and cerebrovascular diseases (all *p* < 0.001).

By contrast, gender was not independently associated with age (β = +1.57 years, *p* = 0.287), and type of ambulatory visit showed no statistically significant independent association with age (all *p* > 0.05).

## 4. Discussion

This single-center epidemiological study provides a comprehensive overview of the clinical workload and demographic structure of ambulatory neurological care in Bulgaria, highlighting marked age-related patterns, diagnostic concentration, and structured service utilization. The findings contribute to the limited body of ambulatory neurology data from Eastern Europe and offer insights relevant to healthcare planning in aging populations. While single-center studies may face constraints regarding external validity, the present study offers significant scientific value by ensuring high methodological homogeneity and rigorous control over confounding variables. By conducting the research within a single institution, we effectively eliminate inter-center variability—such as differences in clinical protocols, equipment calibration, and staff expertise—which often complicates the interpretation of multi-center data. This focused environment allows for collecting high-granularity data and nuanced clinical observations that are frequently lost in larger, logistically complex trials. Consequently, these findings provide a robust proof of concept and establish a reliable foundation for hypothesis generation, serving as a necessary precursor to future large-scale randomized controlled investigations.

### 4.1. Demographic and Epidemiological Patterns

The study population was characterized by a predominance of middle-aged and older adults, with a mean age of 56.9 years and a median of 59 years. This age distribution is consistent with previous reports indicating that neurological disorders disproportionately affect older populations and account for a substantial share of ambulatory healthcare utilization [1,2]. The observed female predominance (55%) aligns with prior studies demonstrating higher ambulatory care use among women, particularly for chronic neurological and pain-related conditions [6,7].

Importantly, gender was not independently associated with age or visit type in multivariable analysis, suggesting that the observed female predominance reflects healthcare-seeking behavior rather than structural differences in service provision. This finding supports previous evidence that gender-related disparities in neurology ambulatory settings are more pronounced in utilization rates than in clinical pathways [8].

### 4.2. Diagnostic Concentration and Service Structure

A key finding of this study is the pronounced concentration of ambulatory visits within a limited number of diagnostic groups, with spine-related disorders accounting for over 40% of visits. Such diagnostic clustering has been reported in other ambulatory neurology cohorts and is typically attributed to the high prevalence of chronic, non-acute neurological conditions requiring repeated follow-up, including cerebrovascular disease, headache disorders, and degenerative conditions [3,4].

Similarly, the dominance of a single visit type suggests a standardized ambulatory care model, likely centered on longitudinal management rather than episodic or acute neurological assessment. This pattern is consistent with healthcare systems facing increasing chronic disease clinical workload and limited inpatient capacity, where ambulatory care serves as the primary setting for ongoing neurological management [4].

### 4.3. Age Stratification Across Diagnostic Categories

Age differed significantly across diagnostic groups, with a moderate-to-large effect size (η^2^ ≈ 0.19), indicating that nearly one-fifth of age variability was explained by diagnostic category alone. Post hoc analysis confirmed substantial heterogeneity, while multivariable linear regression demonstrated that diagnostic group remained independently associated with age after adjustment for gender and visit type.

These findings reflect well-established epidemiological trends in neurology, where certain disorders exhibit strong age dependence, such as neurodegenerative diseases and cerebrovascular conditions, while others are more prevalent in younger or working-age populations [1,5]. The persistence of these associations in adjusted models underscores diagnosis as the primary driver of age-related patterns in ambulatory neurology care.

### 4.4. Multivariable Model Performance and Assumptions

The multivariable linear regression model demonstrated good overall performance (adjusted R^2^ = 0.18–0.20), which is comparable to or exceeds the explanatory power reported in similar ambulatory epidemiological studies [9]. Model diagnostics indicated acceptable adherence to linear regression assumptions. While minor deviations from normality were observed in residual distributions, these are expected in large clinical datasets and are unlikely to materially affect coefficient estimates or inference [10]. No meaningful heteroscedasticity or multi-collinearity was detected.

### 4.5. Implications for Ambulatory Neurology Care

From a health systems perspective, the findings highlight the central role of ambulatory neurology services in managing the clinical workload of age-related neurological disease. The strong diagnostic concentration suggests that targeted ambulatory pathways, disease-specific clinics, and integrated chronic care models may offer substantial efficiency gains. Furthermore, the lack of independent associations between age and visit type implies that current service structures are applied uniformly across demographic strata, which may facilitate equitable access but also warrants evaluation for age-specific optimization.

### 4.6. Strengths and Limitations

Despite its focused scope, a single-center, one-year snapshot was selected as an appropriate and essential first step to establish a baseline mapping of service utilization in a setting where granular, encounter-level data are frequently lacking. This approach ensured high methodological feasibility and data integrity, allowing for an in-depth analysis of clinical workload distribution without the confounding variability of inter-institutional protocols. This study serves as a critical hypothesis-generating tool, identifying specific diagnostic concentrations and follow-up gaps that warrant further investigation. To enhance the external validity of these findings, future multi-center longitudinal studies are needed to track patient trajectories over time and assess the consistency of these utilization patterns across different geographic regions and levels of healthcare facility.

The primary strength of this study is its ability to capture real-world utilization patterns within a specialized outpatient setting, providing a granular view of how systemic factors—such as referral pathways and administrative mandates—shape clinical activity. By analyzing the clinical workload through the lens of healthcare organization, this work offers a rare empirical mapping of patient flow in a post-transition Eastern European context, which is often underrepresented in health services research.

Several limitations must be acknowledged. First, as a single-center study, the findings reflect the specific clinician practice patterns and local organizational structures of a single institution, which may limit their generalizability (external validity) to other regions or facility types. Second, the data represent utilization volume and should not be interpreted as epidemiological prevalence or incidence. The high concentration of certain conditions, such as spine-related disorders, likely reflects access barriers and triage pressures at the primary care level rather than the actual frequency of these diseases in the general population. Finally, because in the study we relied on administrative and clinical records, we could not account for patients who sought care outside the public insurance framework (out-of-pocket), potentially underestimating the total demand for neurological services in the region.

### 4.7. Study Setting and Regional Context

The Bulgarian healthcare system presents a unique organizational framework that distinguishes its utilization data from typical Western European or North American datasets. Operating under a compulsory social health insurance model managed by the National Health Insurance Fund (NHIF), the system utilizes a strict ‘gatekeeper’ mechanism. Access to specialized neurological care is primarily regulated through a formal referral system (Direction No. 3), meaning that the patient cohort in this study represents a pre-filtered population whose needs exceeded the scope of primary care. A defining feature of this setting is the formalized dispensary system—a structured longitudinal monitoring framework for chronic conditions. Unlike many Western models where follow-up care is often ad hoc or decentralized, the Bulgarian model mandates standardized intervals for specialist consultations based on specific diagnostic codes. Furthermore, the system operates under rigid monthly referral quotas, which creates a competitive environment for specialist access. Consequently, these data provide a high-value ‘snapshot’ of how a middle-income Eastern European country prioritizes specialized resources, offering critical insights into patient pathways and service organization.

## 5. Conclusions

In this study, we provide a comprehensive analysis of the outpatient neurological landscape within an underrepresented health system setting, offering critical insights into the operational dynamics of the Bulgarian ambulatory model. By explicitly differentiating between acute diagnostic (ambulatory) and chronic follow-up (dispensary) encounters, our findings reveal a healthcare environment characterized by a high volume of primary consultations and a significant concentration of spine-related pathology. The innovation of this work lies in its ability to bridge the gap between clinical neurology and health system management. We demonstrate that the current outpatient clinical workload is dominated by musculoskeletal neurological conditions, accounting for over 40% of the clinical workload, which suggests a pressing need for specialized pain management protocols and targeted resource allocation within the primary neurology sector. Furthermore, the identified distribution of visit types—marked by a predominant reliance on primary consultative examinations (65.4%)—underscores a high-turnover system that demands streamlined triage and enhanced continuity of care strategies. Ultimately, this research advances existing knowledge by providing a methodological and empirical baseline for Eastern European outpatient care. It serves as a necessary precursor to large-scale, multi-center studies aimed at optimizing patient pathways and improving the long-term management of chronic neurological morbidity in a public insurance-driven framework.

## Figures and Tables

**Table 1:** Baseline characteristics of the ambulatory visits (N = 518).

Characteristic	n	%
Gender		
Male	231	44.6
Female	287	55.4
Age (years)		
Mean ± SD	56.9 ± 18.4	
Median (IQR)	59.0 (42.0–73.0)	
Age group (years)		
≤20	7	1.4
21–30	56	10.8
31–40	55	10.6
41–50	72	13.9
51–60	83	16.0
61–70	78	15.1
71–80	137	26.4
81–90	30	5.8
Type of visit		
Primary consultative examination	339	65.4
Secondary consultative examination	96	18.5
Primary dispensary examination	35	6.8
Secondary dispensary examination	48	9.3
Season		
Winter	112	21.6
Spring	160	30.9
Summer	138	26.6
Autumn	108	20.8

**Table 2:** One-way ANOVA for age differences across diagnostic groups.

Variable	F Statistic	Degrees of Freedom	*p*-Value	Effect Size (η^2^)
Age across diagnostic groups	13.02	9.508	<0.001	0.19

**Table 3:** Post hoc Tukey HSD comparisons for age differences between selected diagnostic groups.

Comparison	Mean Difference (Years)	95% CI	Adjusted *p*-Value
Neurodegenerative vs. Spine-related disorders	+26.5	14.2 to 38.8	<0.001
Cerebrovascular vs. Spine-related disorders	+18.7	9.4 to 28.0	<0.001
Cerebrovascular vs. Headache and pain syndromes	+17.2	7.9 to 26.5	<0.001
Neurodegenerative vs. Peripheral nervous system disorders	+20.3	8.7 to 31.9	<0.001
Movement disorders vs. Spine-related disorders	+11.4	2.8 to 20.0	0.004
Epilepsy and seizures vs. Neurodegenerative disorders	−15.1	−27.8 to −2.4	0.012
Vestibular disorders vs. Headache and pain syndromes	+9.6	1.7 to 17.5	0.009

**Table 4:** Multivariable linear regression analysis with age as the dependent variable.

Predictor	β Coefficient (Years)	95% CI	Standardized β	*p*-Value
Female sex	+1.57	−1.31 to 4.45	0.04	0.287
Secondary consultative examination	+0.94	−3.12 to 5.01	0.03	0.648
Primary dispensary examination	+2.83	−4.62 to 10.28	0.05	0.456
Secondary dispensary examination	+3.91	−2.88 to 10.70	0.07	0.259
Spine-related disorders	−12.4	−18.1 to −6.7	−0.31	<0.001
Headache and pain syndromes	−18.7	−26.5 to −10.9	−0.28	<0.001
Peripheral nervous system disorders	−7.8	−14.6 to −1.0	−0.14	0.024
Vestibular disorders	−5.9	−12.2 to 0.4	−0.09	0.067
Epilepsy and seizures	−9.6	−17.8 to −1.4	−0.16	0.021
Neurodegenerative disorders	+14.8	5.7 to 23.9	+0.18	<0.001
Cerebrovascular diseases	+11.2	4.6 to 17.8	+0.19	<0.001

## Data Availability

The data presented in this study are available on request from the corresponding author due to privacy.

## Abbreviations

The following abbreviations are used in this manuscript:

NHIF	National Health Insurance Fund
ICD-10	Classification of Diseases and Related Health Problems 10th Revision
EHRs	Electronic health records
SDs	Standard Deviations
ANOVA	One-Way Analysis of Variance
HSD	Honestly Significant Difference
CI	Confidence Interval

## References

[1] Feigin V.L., Nichols E., Alam T., Bannick M.S., Beghi E., Blake N., Culpepper W.J., Dorsey E.R., Elbaz A., Ellenbogen R.G. (2019). Global, regional, and national burden of neurological disorders, 1990–2016: A systematic analysis for the Global Burden of Disease Study 2016. Lancet Neurol..

[2] Vos T., Lim S.S., Abbafati C., Abbas K.M., Abbasi M., Abbasifard M., Abbasi-Kangevari M., Abbastabar H., Abd-Allah F., Abdelalim A. (2020). Global burden of 369 diseases and injuries in 204 countries and territories, 1990–2019: A systematic analysis for the Global Burden of Disease Study 2019. Lancet.

[3] Stone J., Carson A., Duncan R., Roberts R., Warlow C., Hibberd C., Coleman R., Cull R., Murray G., Pelosi A. (2010). Who is referred to neurology clinics?—The diagnoses made in 3781 new patients. Clin. Neurol. Neurosurg..

[4] Busse R., Blümel M., Scheller-Kreinsen D., Zentner A. (2010). Tackling Chronic Disease in Europe: Strategies, Interventions and Challenges.

[5] Prince M.J., Wimo A., Guerchet M.M., Ali G.C., Wu Y.-T., Prina M. (2015). World Alzheimer Report 2015: The Global Impact of Dementia—An Analysis of Prevalence, Incidence, Cost and Trends.

[6] Thompson A.E., Anisimowicz Y., Miedema B., Hogg W., Wodchis W.P., Aubrey-Bassler K. (2016). The influence of gender and other patient characteristics on health care-seeking behaviour: A QUALICOPC study. BMC Fam. Pract..

[7] Steiner T.J., Stovner L.J., Katsarava Z., Lainez J.M., Lampl C., Lantéri-Minet M., Rastenyte D., Ruiz de la Torre E., Tassorelli C., Barré J. (2014). The impact of headache in Europe: Principal results of the Eurolight project. J. Headache Pain.

[8] Pinkhasov R.M., Wong J., Kashanian J., Lee M., Samadi D.B., Pinkhasov M.M., Shabsigh R. (2010). Are men shortchanged on health? Perspective on health care utilization and health risk behavior in men and women in the United States. Int. J. Clin. Pract..

[9] McKee M., Healy J. (2002). Hospitals in a Changing Europe.

[10] Lumley T., Diehr P., Emerson S., Chen L. (2002). The importance of the normality assumption in large public health data sets. Annu. Rev. Public Health.

